# Using Administrative Claims to Model Health-Related Behaviors: Measures of Screening and Medication Adherence

**DOI:** 10.1093/geroni/igab046.1065

**Published:** 2021-12-17

**Authors:** Arseniy Yashkin

**Affiliations:** Duke University, Morrisville, North Carolina, United States

## Abstract

We demonstrate how administrative claims records can be used to model certain behavioral patterns and associated health effects. The inability of administrative claims, which are in essence a billing record, to account for differences in behavior is a major limitation of such data which usually requires an externally linked source to overcome. However, for certain diseases, for which well-defined and accepted guidelines on screening and medication use exist, the claims themselves can provide a way for modeling health-related behavior. A practical application to screening and medication adherence for type ii diabetes mellitus is presented. Diverse methods of the calculation of such indexes with their pros, cons and variation in identified effects are discussed and demonstrated using results based on administrative claims drawn from a 5% sample of Medicare beneficiaries.

